# Changes of Vestibular Symptoms in Menière's Disease After Triple Semicircular Canal Occlusion: A Long-Term Follow-Up Study

**DOI:** 10.3389/fneur.2022.797699

**Published:** 2022-02-04

**Authors:** Yumeng Jiang, Maoxiang Xu, Qingxiu Yao, Zhuangzhuang Li, Yaqin Wu, Zhengnong Chen, Dongzhen Yu, Haibo Shi, Shankai Yin

**Affiliations:** ^1^Department of Otolaryngology-Head and Neck Surgery, Shanghai Jiao Tong University Affiliated Sixth People's Hospital, Shanghai, China; ^2^Otolaryngology Institute of Shanghai Jiao Tong University, Shanghai, China; ^3^Shanghai Key Laboratory of Sleep Disordered Breathing, Shanghai, China

**Keywords:** vestibular symptoms, triple semicircular canal occlusion, vestibular nerve section, Menière's disease, clinical benefit

## Abstract

**Background:**

The clinical efficacy of triple semicircular canal occlusion (TSCO) and vestibular nerve resection (VNS) for patients with Ménière's disease has been unclear.

**Objective:**

To explore changes in vestibular symptoms after TSCO and its advantages compared to the classical operation of VNS in patients with Menière's disease.

**Methods:**

In total, 36 patients with Menière's disease performed TSCO or VNS at Shanghai Jiao Tong University Affiliated Sixth People's Hospital, China from May 2005 to July 2021, and all of them were enrolled in our study. Twelve of them underwent TSCO, 23 underwent VNS, and 1 had both treatments. We compared the demographic parameters, clinical symptoms, and selected test results between the two surgical methods. Ten patients each who underwent TSCO and VNS completed the follow-up. We collected and compared data pertaining to changes in vestibular symptoms.

**Results:**

No significant difference in demographic parameters, clinical symptoms, or auditory or vestibular test results was detected between the two groups preoperatively. The TSCO group with vertigo as the main complaint experienced less residual paroxysmal dizziness after surgery than the VNS group (*P* = 0.020). Also, 57% of the patients in the VNS group had unsteadiness after surgery, while no such problems were reported in the TSCO group (*P* = 0.025).

**Conclusions:**

Our study shows that TSCO controls vertigo in most Menière's disease patients, and also has the advantage of lower rates of postoperative paroxysmal dizziness and unsteadiness than VNS. Thus, TSCO may be an effective surgery for refractory Menière's disease.

## Introduction

Menière's disease is an inner ear disorder characterized by two or more episodes of vertigo associated with fluctuating low- and medium-frequency hearing loss (HL), tinnitus, and aural fullness. The duration of vertigo attacks is between 20 min and 12 h (1). An epidemiological survey in Japan showed that 50–200 out of 100,000 adults suffer from Menière's disease, which mostly occurs in people aged 40–60 years (2, 3). The disease is also characterized by an impaired quality of life (4) and can lead to restricted activity due to refractory vertigo (5).

Both medical and surgical interventions are used in the treatment of Menière's disease, to control vertigo attacks and preserve hearing and vestibular function (6). A series of relatively reliable treatments has been developed for Menière's disease (3). Improving lifestyle and exercising more is the initial treatment step. Previous studies reported that a low-salt diet free from caffeine can help balance the ion concentration in the endolymph of the inner ear (7). The next step of treatment is to reduce the number of attacks and relieve vertigo and dizziness symptoms *via* medications including betahistine, antihistamines, benzodiazepines, ginkgo biloba extract, and corticosteroids (8); diuretics may also be tried to reduce endolymphatic hydrops (9). Most of the symptoms can be stably controlled by medicine, although 15–40% of patients suffer disabling refractory vertigo (7).

For those living with serious vertigo attacks for years, and in whom lifestyle changes and medical therapy have been tried unsuccessfully various surgical treatments are available according to the patient's hearing status (3). Endolymphatic sac surgery (ESS), labyrinthectomy, and vestibular nerve resection (VNS) were recommended by the AAO-HNS clinical guidelines as early as 1985 (10). Dandy performed the first vestibular nerve section in 1942 and advocated selective nerve section for intractable Menière's disease thereafter (11). In 1990 Parnes and McClure described posterior semicircular canal occlusion for benign paroxysmal positional vertigo (12). The purpose is to eliminate the movement endolymph in the canals, so as to eliminate the vertigo attack caused by specific head movement in patients with BPPV. We hypothesize the pathophysiology of Menière's disease is that excessive accumulation of endolymph can cause bulk endolymph movement and episodes of spinning vertigo, and this can be improved by semicircular canal obstruction. Triple semicircular canal occlusion (TSCO) for Menière's disease was advocated by Yin et al. (13), and has been performed in China and elsewhere with good vertigo control and preservation of hearing (14, 15).

However, due to the lack of comparison of clinical efficacy between different surgical methods, it is difficult to determine the optimal approach for refractory Menière's disease (16). Therefore, the aim of the present study is to evaluate the effectiveness of TSCO to control vertigo attacks over the long term and improve quality of life compared to traditional VNS.

## Methods

### Study Design

A retrospective study was performed of patients with Menière's disease in Shanghai Jiao Tong University Affiliated Sixth People's Hospital, China from May 2005 to July 2021. The indications for surgery were a pure-tone average of >70 dB Hearing Level (HL) (according to the AAO-HNS clinical guidelines), failure of previous medical therapy applied over at least 6 months, and a strong desire for surgery. The exclusion criteria included a history of any other neurogenic disease, vestibular neuritis, brain tumor, vestibular migraine, BPPV, or any other disease that can cause vertigo.

Before surgery, all patients underwent a series of auditory and vestibular evaluations, including pure-tone audiometry, tympanometry, distortion product otoacoustic emission and glycerol test. Tympanometry is used to test the condition of the middle ear by creating air pressure in the ear canal. Distortion product otoacoustic emission, otoacoustic emission evoked by two pure tones, reflect the integrity of outer hair cell and the function of cochlea (17). The glycerol test was regarded as positive in the audiometry if the pure tone threshold improved at least 15 dB at minimum 3 frequencies after ingesting glycerol (18). Patients seen within the last 3 years also underwent radiological examination, including temporal bone computed tomography and magnetic resonance imaging, and intratympanic gadolinium-enhanced inner-ear magnetic resonance imaging.

Changes of vestibular symptoms were evaluated by telephone follow-up, which inquired about attacks of vertigo after surgery and other symptoms. The specific questions are as follows: Would you please describe the change of your vestibular symptoms during these year after surgery; Was there any vertigo attacked again after surgery and when? Were there any symptoms such as dizziness and unsteadiness appeared? When and how it happened; How about your hearing condition? We calculated the average number of attacks for each ear. In July 2021, we administered four questionnaires by telephone to each TSCO patient: the Dizziness Handicap Inventory (DHI), Activities-Specific Balance Confidence Scale (ABC), Tinnitus Handicap Inventory (THI), and Visual Analog Scale (VAS).

The study was designed and executed in accordance with the recommendations of the Ethics Committee of Shanghai Jiao Tong University Affiliated Sixth People's Hospital (2020 KY 004).

### Patients and Outcome Measures

In the past 16 years, 99 Menière's disease patients underwent surgery, including TSCO, VNS, and ESS, at Shanghai Jiao Tong University Affiliated Sixth People's Hospital. Of these 99 patients, 36 patients underwent TSCO or VNS and all were included in our retrospective study. Ten patients per surgical method who completed follow-up were included and analyzed in this study in terms of the curative effect of surgery, The remaining 16 patients were unable to be contacted or had not returned to our hospital.

Vestibular symptoms, including vertigo, dizziness, and unsteadiness, were assessed before and after surgery (19). The primary outcome was vertigo control as determined by the number of vertigo episodes. According to the American Academy of Otolaryngology–Head and Neck Surgery guidelines (1), the patient's vertigo control status was determined by comparison of the frequency of vertigo attacks between 6 months before and 18–24 months after surgery. Vertigo control was expressed numerically (number of attacks after treatment divided by the number prior to the treatment) as follows; 0 = A, complete control; 1–41 = B, substantial control; 41–80 = C, limited control; 81–120 = D, insignificant control; and > 120 = E, no control. The postoperative data of all patients were collected by telephone in July 2021. Dizziness and unsteadiness were secondary outcomes. In the TSCO group, the DHI, ABC, and VAS scores were used to analyze secondary outcomes by evaluating vertigo, dizziness and balance; the THI score was used to evaluate the severity of tinnitus. The DHI, a questionnaire exploring the self-perceived handicapping effects of vestibular disease consists of three parts (DHI-P, -E and -F) evaluated separately (20). Visual analog scale (VAS) was administered *via* telephone to measure symptoms or conditions that are not captured by the other measures. We asked the patient to rate their current symptom level on a scale from 0 to 10, with 10 being the patient's symptom at the time when he was most affected by Menière's disease before surgery, and 0 being the patient's symptom when completely healthy. For the VNS group, clinical information including vertigo attacks, unsteadiness, HL, and tinnitus was collected by telephone or outpatient follow-up. All of the information was collected in July 2021.

Hearing (clinical stage) was assessed according to the pure-tone average at frequencies of 0.5, 1, and 2 kHz; this also informed the surgical method, along with clinical guidelines. The hearing of patients was categorized as follows: 0–25 dB HL = stage 1; 26–40 dB HL = stage 2; 41–70 dB HL = stage 3; and > 70 dB HL = stage 4.

### Surgical Protocol for TSCO

**Figure 1:**
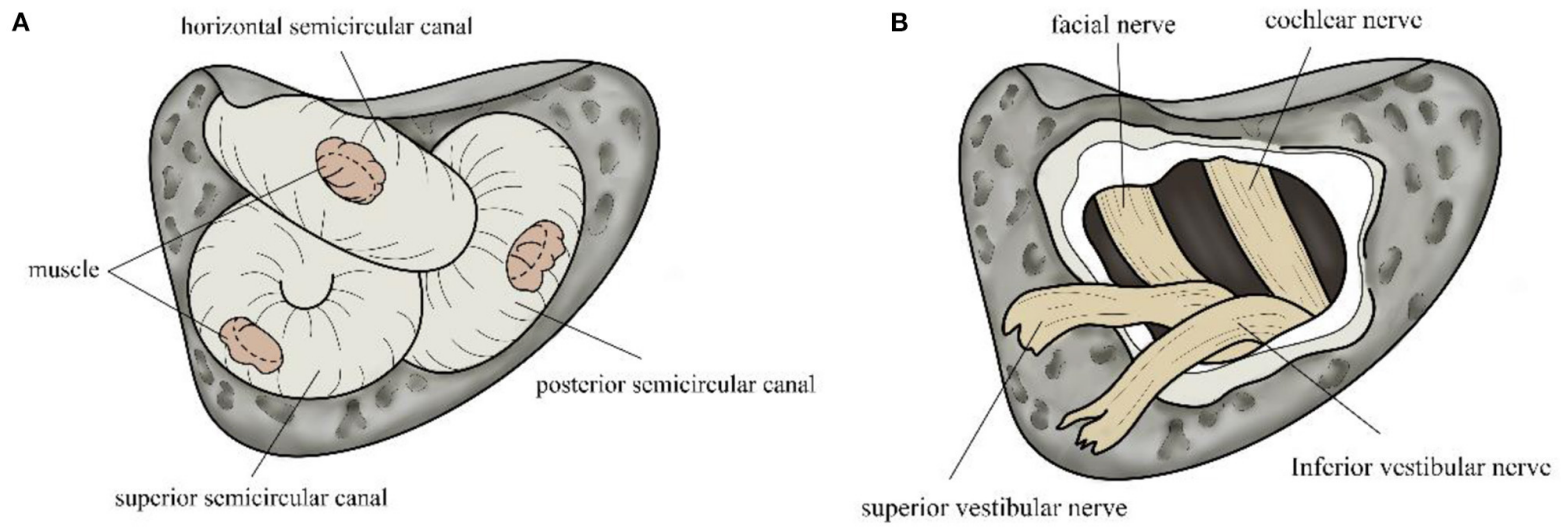
Diagram of triple semicircular canal occlusion and vestibular nerve resection. **(A)** Muscle tissue blocks the three semicircular canals, which are clearly contoured. **(B)** The vestibular is cut off after being separated from the cochlear nerve.

Yin et al. (13, 14, 21) were the first to modify SCO, which was originally performed in patients with BPPV, and apply it to Menière's disease in 2004. After standard mastoidectomy, three semicircular canals are contoured and a bone island with a diameter of approximately 1 mm is drilled in the non-ampullary arm of each canal. Then, great care is taken while prying off each bone island with a hook, and each canal is completely occluded with a piece of muscle or fascia tissue (Figure 1A). Next, the access area is covered by a piece of fascia with some bone dust sprayed on the muscle tissue. Finally, a piece of gelatin sponge is placed on the surgical site.

### Surgical Protocol for VNS

Retrolabyrinthine and retrosigmoid approaches were used in the VNS group (22). The difference between these approaches lies in the location in which the dura mater is exposed. The first approach contours the mastoid and opens the internal auditory canal to 270°. The second approach opens a bone window approximately 3 cm × 4 cm in size at the junction of the parietal, occipital, and temporal bones. Then, the dura mater is opened, and cerebrospinal fluid released. The cerebellum is gently pressed to expose the cerebellopontine angle and separate the vestibular and cochlear nerves. The vestibular nerve is then cut. After cleaning the cavity the surgical site is filled with hemostatic silk and gelatin sponge (23) (Figure 1B).

### Statistical Analysis

Statistical analysis was performed with SPSS software (version 25.0; IBM Corp., Armonk, NY, USA). The normality of continuous variables was assessed with the Shapiro–Wilk test. We express normally distributed variables as mean ± standard deviation, and compared the two groups using independent sample *t*-tests; we express non-normally distributed variables as mean ± standard deviation, and used nonparametric tests (Mann–Whitney U test) to compare the groups. We express categorical variables as frequency or percentage and used the chi-square test to compare the groups. We considered *P* < 0.05 statistically significant.

## Results

### Clinical Symptoms and Tests

During the past 16 years, 99 Menière's disease patients underwent surgery in Shanghai Jiao Tong University Affiliated Sixth People's Hospital, of which 12 and 23 underwent TSCO and VNS, respectively (one patient underwent VNS in 2005 and TSCO in 2017), and were included in our retrospective study. There were no statistically significant differences between the two groups in demographic parameters, including gender and age at surgery, or clinical symptoms, including duration of vertigo attack, number of years of attacks, number of vertigo spells in the 6 months before surgery, worst hearing level, number of years of HL, tinnitus, or affected side (all *P* > 0.05; Table 1). Similarly, no significant differences between the groups were detected in the results of auditory tests before surgery, including tympanometry, distortion product otoacoustic emission and glycerol tests.

**Table 1:** Patient demographics, symptoms, and results of tests before surgery.

	Total (***N*** = 35*)	**TSCO (***N*** = 12)**	**VNS (***N*** = 23)**	***P*** **-value**
**Gender**				
Male (*n*/%)	14(40%)	5(42%)	9(39%)	0.884
Female (*n*/%)	21(60%)	7(58%)	14(61%)	
Age at surgery (years)	53.46 ± 9.77	53.58 ± 10.92	53.39 ± 9.38	0.957
**Side**				
Right (*n*/%)	17 (49%)	7 (58%)	10 (43%)	0.404
Left (*n*/%)	18 (51%)	5 (42%)	13 (57%)	
**Vertigo**				
Duration (years)	7.55 ± 7.12	6.89 ± 3.59	7.91 ± 8.52	0.68
Average duration of vertigo episode(h)	5.48 ± 6.58	2.78 ± 3.13	4.90 ± 5.81	0.65
Number of spells 6 months before surgery (*n*)	15.26 ± 10.93	16.25 ± 11.68	14.73 ± 10.74	0.71
**Hearing loss**				
Worst hearing (dB)	67.26 ± 20.61	69.58 ± 13.37	66.04 ± 23.71	0.637
Duration (years)	6.75 ± 6.92	5.53 ± 3.32	7.48 ± 8.38	0.99
**Tinnitus**				
Duration (years)	8.42 ± 10.27	8.56 ± 13.60	8.34 ± 8.29	0.49
**Test**				
**Tympanogram**				
A (*n*/%)	21 (91%)	10 (91%)	11 (92%)	0.949
As (*n*/%)	2 (9%)	1 (9%)	1 (8%)	
**Distortion product otoacoustic emission (** ***n*** **/%)**				
Present	1(6%)	0(0%)	1(12.5%)	0.274
Absent	16(94%)	9(100%)	7(87.5%)	
**Glycerol test (** ***n*** **/%)**				
Positive	10(77%)	3(60%)	7(87.5%)	0.252
Negative	3(23%)	2(40%)	1(12.5%)	

* *One patient who underwent both triple semicircular canal occlusion and vestibular nerve resection was excluded from the analyses*.

### Vertigo Control

**Figure 2:**
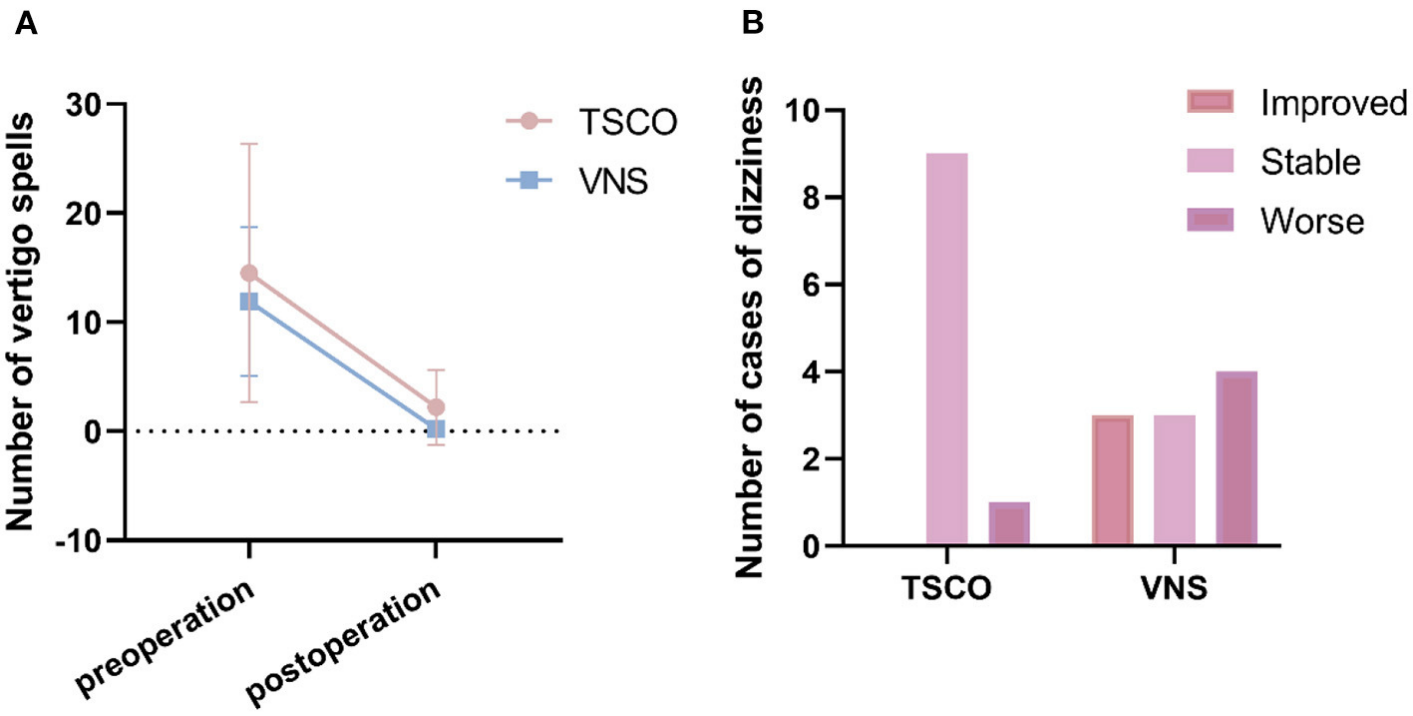
Incidence of postoperative vertigo and paroxysmal dizziness. **(A)** Vertigo spells were controlled by both triple semicircular canal occlusion (TSCO) and vestibular nerve resection (VNS), and there was no significant difference in incidence between the two groups. **(B)** Bar chart comparing the numbers of postoperative cases of paroxysmal dizziness between the two groups.

According to the American Academy of Otolaryngology–Head and Neck Surgery guidelines (1), patients who underwent surgery more than 24 months ago (*N* = 4 and 10 in the TSCO and VNS groups, respectively) were analyzed in terms of post-operation vertigo within 2 years; 100% of the patients in the TSCO group achieved complete control of vertigo, compared to 90% in the VNS group (10% achieved substantial control). During the entire follow-up period, four patients in the TSCO group and seven in the VNS group had no vertigo attacks, and three patients in the VNS group had a vertigo attack. There was no significant difference in number of postoperative vertigo episodes in 24 months between the two surgical methods using independent sample *t*-tests (*P* = 0.839). In brief, the surgical methods had similar efficacy for vertigo attack control (number of attacks after treatment divided by the number prior to the treatment) for most patients in two group got an A control in 2 years after surgery using chi-square test (*P* = 0.512; Table 2; Figure 2A). Six patients in the TSCO group underwent surgery <24 months before this study was completed, and 50% of them had no vertigo attacks after surgery. Although the other 50% of these patients had vertigo attacks, their frequency was much lower than before TSCO. However, one patient whose main complaint was severe dizziness rather than vertigo had no benefit after surgery. We also counted the postoperative vertigo control of the two groups regardless of the postoperative time. The comparison of vertigo control between the two groups was unchanged (*all P* > *0.05*; Table 3).

**Table 2:** Vertigo control after surgery in patients who performed surgery more than 24 months ago.

	**Total (***N*** = 14)**	TSCO (***N*** = 4*)	**VNS (***N*** = 10)**	***P*** **-value**
Number of vertigo episodes in 24 months after surgery	0.07 ± 0.28	0.00 ± 0.00	0.10 ± 0.32	0.839
**Class**				
A	13 (93%)	4 (100%)	9 (90%)	0.512
B	1 (7%)	0 (0%)	1 (10%)	

* *Four of 10 patients in the triple semicircular canal occlusion group were operated on more than 24 months ago*.

**Table 3:** Vertigo control after surgery in all follow-up patients.

	**Total (***N*** = 20)**	**TSCO (***N*** = 10)**	**VNS (***N*** = 10)**	***P*** **-value**
Number of vertigo episodes	1.15 ± 2.60	2.20 ± 3.43	0.10 ± 0.32	0.218
**Class**				
A	15 (75%)	6 (60%)	9 (90%)	0.273
B	4 (20%)	3 (30%)	1 (10%)	
D	1 (5%)	1 (10%)	0 (0%)	

Ten patients in the TSCO group completed four questionnaires *via* telephone during follow-up. Based on the DHI results, we evaluated the patients' quality of life physically, emotionally, and functionally; a large group difference was found in all three domains (*P* = 0.022, 0.025, and 0.013, respectively). The scores were significantly lower in patients who underwent TSCO more than 24 months ago compared to those operated on more recently. However, no significant differences were found in the ABC, THI, or VAS scores (all *P* > 0.05; Table 4).

**Table 4:** Dizziness handicap inventory, activities-specific balance confidence scale, tinnitus handicap inventory, and visual analog scale scores of the triple semicircular canal occlusion patients.

	**Total (***N*** = 10)**	**Operated on within the last 24 months (***N*** = 6)**	**Operated on more than 24 months ago (***N*** = 4)**	***P*** **-value**
DHI-P	7.33 ± 8.60	12.40 ± 8.53	1.00 ± 2.00	0.022
DHI-E	11.33 ± 12.73	19.20 ± 12.13	1.50 ± 1.92	0.025
DHI-F	14.67 ± 17.75	26.00 ± 16.37	0.50 ± 1.00	0.013
ABC (0–10)	2.56 ± 3.97	3.20 ± 4.60	1.75 ± 3.50	0.662
THI (0–100)	12.44 ± 20.61	12.40 ± 25.55	12.50 ± 16.20	0.441
VAS (1–10)	3.50 ± 3.32	5.20 ± 3.56	1.38 ± 1.25	0.082

### Postoperative Paroxysmal Dizziness and Unsteadiness

Postoperative problems requiring attention include paroxysmal dizziness (sensation of disturbed or impaired spatial orientation without a distorted sense of motion), unsteadiness (postural instability when sitting, standing, or walking (19)), and vertigo recurrence. As one of the symptoms of vestibular function disorder dizziness may coexist with vertigo in Menière's disease. In terms of paroxysmal dizziness, different patients have different conditions. Using the number of patients with postoperative paroxysmal dizziness to analyze was improper for losing the individual characteristics of patients. All patients were encouraged to exercise and no specific vestibular physical therapy was performed. After surgery we scored the symptoms of our patients as follows: 1, improved: dizziness had occurred before but disappeared after the operation; 2, stable: dizziness existed before and after the operation (or asymptomatic patients); or 3, worse: no dizziness previously, but dizziness onset after the operation. The data indicated that TSCO was significantly better than VNS in reducing dizziness (*P* = 0.020; Table 5; Figure 2B). unsteadiness, which is a symptom of vestibular dysfunction, only occurred after VNS (*P* = 0.025).

**Table 5:** Comparison of postoperative symptoms between the two groups.

	**Total (***N*** = 20)**	**TSCO (***N*** = 10)**	**VNS (***N*** = 10)**	***P*** **-value**
**Dizziness**				
Improved (*n*/%)	3(15%)	0 (0%)	3 (30%)	0.020
Stable (*n*/%)	12 (60%)	9 (90%)	3 (30%)	
Worse (*n*/%)	5 (25%)	1 (10%)	4 (40%)	
**Unsteadiness (** ***n*** **/%)**	4 (18%)	0 (0%)	4 (36%)	0.025

### Special Case

One patient in our study underwent both TSCO and VNS. He underwent ESS for recurrent vertigo in 2002 but it recurred. Therefore, in 2005, he underwent VNS and vertigo control was achieved. However, dizziness and unsteadiness occurred after this surgery and lasted for 10 years. In 2016, his vertigo recurred with attacks lasting 2 h. After undergoing TSCO in 2017, his vertigo and dizziness disappeared completely, but functional compensation for the unsteadiness was difficult to achieve.

## Discussion

In this retrospective trial, we found no significant difference between TSCO and VNS in terms of the ability to control vertigo. At 24 months after surgery, all patients in the TSCO group achieved class A vertigo control, whereas there were nine cases of class A vertigo, and 1 of class B, in the VNS group. The results confirm the efficacy of the two surgical methods for controlling vertigo symptoms. However, other vestibular symptoms including dizziness and unsteadiness were less common among TSCO than VNS patients. It is plausible that the vestibular nerve on the healthy side cannot completely compensate for the balance disorders caused by VNS on the affected side. Our study also showed that the DHI scores of patients more than 24 months after TSCO were significantly lower than those within 24 months, reflecting a change in patient emotional, physical, and functional quality of life. Vestibular function recovery in TSCO patients takes time.

Fan et al. (24) reported a 100% success rate for vertigo control after TSCO, with 81.6% and 18.4% of patients achieving complete and partial control, respectively, after 2 years (25). Setty et al. (26) reported that vertigo was controlled in 97.7% of Menière's disease patients after VNS. Similarly high rates of control of vertigo have also been reported in other studies. However, researchers often ignore postoperative symptoms surgeons can only choose the most effective surgical method by studying the relationships among vertigo and dizziness, unsteadiness, and vestibular disorder.

Surgical ablative therapies are considered an option for patients with active Menière's disease who have failed less definitive treatments and have unusable hearing (3). For nearly a century the VNS operation has been used to treat intractable Menière's disease (since the 1920s (11)). VNS controls vertigo by cutting the vestibular nerve, which sacrifices vestibular function on the lesioned side (11). This could explain why unsteadiness, dizziness attacks, and residual dizziness occur more often in VNS than TSCO patients, with the former group correspondingly showing larger changes in postoperative quality of life. By contrast, TSCO only blocks endolymph flow in three semicircular canals, which decreases vestibular function only on the surgical side (13). Overall, TSCO is equally effective as VNS for vertigo control, but is associated with a lower rate of dizziness.

A major concern with TSCO is postoperative HL. Stultiens et al. (27) assessed the postoperative hearing of patients with a semicircular canal plug and found a difference from the preoperative hearing level of <10 dB HL at the 6-month follow-up. Their study included stage 1–2 patients. In 2015 Zhang et al. (25) reported a rate of hearing preservation of 69.4% for TSCO. In the present study, the patients who underwent TSCO were mostly in stage 4, so it was difficult to determine whether TSCO was helpful for postoperative hearing recovery and preservation.

VNS is an intracranial operation with potential risks for cerebrospinal fluid leaks and facial nerve injury (28). In our patients none of these occurred. Risk for facial nerve injury can be decreased by a retrosigmoid approach (28). Watertight sutures of meninges, use of mannitol, and raising the head to 30 degrees postoperatively will minimize cerebrospinal fluid leaks.

There are some other surgical approaches available for refractory Menière's disease. According to a survey by the American Academy of Otolaryngology–Head and Neck Surgery Foundation, ESS was the initial surgical intervention for the treatment of Menière's disease in 50% of the respondents (7). Portmann et al. (29) first described ESS in 1927. Saliba et al. (30) reported that the vertigo control rate was 37.5% 4 months after ESS. The main advantage of this surgery is that the hearing is preserved, however, no significant difference in symptom improvement was found between ESS and placebo groups in a study by Bretlau (31), although no reported complications or side effects were found either. Despite this controversy over its effectiveness, ESS is still widely used (32).

Parnes et al. (21) found that blockage of a semicircular canal did not influent the function of the other canal receptors. TSCO, which has the same principle, may preserve some vestibular and hearing function on the side of surgery (13). The result of v-HIT showed a decrease of function of semicircular canals, which increased the threshold of vertigo attack (33). Animal models have indicated that endolymphatic movement caused by excessive accumulation of endolymph will decrease or disappear after semicircular canal obstruction theoretically (13). TSCO exerts its effects as follows. Occlusions partially prevent the endolymph from stimulating the semicircular canals, and displacement of the crista ampullaris (which contributes to rotational vertigo). Also, postoperative disequilibrium is quickly compensated for (34). Our study provides guidance on the surgical method for Menière's disease patients, by demonstrating that TSCO is an alternative choice for vertigo control.

## Limitations

There were several limitations to our study. First, we estimated the effects of surgery *via* pre- and post-operative comparison, which limited the sample size given the lack of postoperative auditory and vestibular evaluation results in some cases. This bias may have affected the accuracy of the analysis. Also, we may have underestimated the rate of postoperative vestibular symptoms given the small sample size, and the long follow-up time might have led to recall bias. Finally, postoperative data for pure-tone audiometry, for example, were incomplete, which limited our audiological evaluation. However, these limitations did not prevent us from describing changes of vestibular symptoms after surgery. Large-scale research using the same pre- and post-operative tests applied in the present study is required.

## Conclusion

The difficulty of treating refractory Menière's disease is well recognized, so selecting the optimal surgical method remains challenging. Our study shows that TSCO controls vertigo in most Menière's disease patients, and also has the advantage of lower rates of postoperative paroxysmal dizziness and unsteadiness than VNS. Thus, TSCO can be considered as an effective surgery for refractory Menière's disease.

## Data Availability Statement

The raw data supporting the conclusions of this article will be made available by the authors, without undue reservation.

## Ethics Statement

The studies involving human participants were reviewed and approved by Shanghai Jiao Tong University Affiliated Sixth People's Hospital (2020 KY 004). Written informed consent for participation was not required for this study in accordance with the national legislation and the institutional requirements.

## Author Contributions

DY and ZC: designed and coordinated the study. YJ, QY, ZL, and MX: analyzed the data and wrote the manuscript. DY, ZC, YW, HS, and SY: performed the surgery. ZC and DY: attests that all listed authors meet authorship criteria and that no others meeting the criteria have been omitted. All authors contributed to the article and approved the submitted version.

## Funding

This work was supported by Shanghai Municipal Education Commission–Gaofeng Clinical Medicine Grant (20191921) and National Key Research and Development Project (2019YFC0119900).

## Conflict of Interest

The authors declare that the research was conducted in the absence of any commercial or financial relationships that could be construed as a potential conflict of interest.

## Publisher's Note

All claims expressed in this article are solely those of the authors and do not necessarily represent those of their affiliated organizations, or those of the publisher, the editors and the reviewers. Any product that may be evaluated in this article, or claim that may be made by its manufacturer, is not guaranteed or endorsed by the publisher.
